# Coronary artery calcium burden, carotid atherosclerotic plaque burden, and myocardial blood flow in patients with end-stage renal disease: A non-invasive imaging study combining PET/CT and 3D ultrasound

**DOI:** 10.1007/s12350-020-02080-w

**Published:** 2020-03-05

**Authors:** Christian Wenning, Alexis Vrachimis, Hermann-Joseph Pavenstädt, Stefan Reuter, Michael Schäfers

**Affiliations:** 1 Department of Nuclear Medicine, University Hospital Münster, Albert-Schweitzer-Campus 1, Building A1, 48149 Münster, Germany; 2 Department of Internal Medicine D, General Internal Medicine and Nephrology, University Hospital Münster, Münster, Germany; 3 European Institute for Molecular Imaging (EIMI), University of Münster, Münster, Germany; 4 DFG EXC 1003 Cluster of Excellence ‘Cells in Motion’, University of Münster, Münster, Germany

**Keywords:** End-stage renal disease, atherosclerosis, plaque burden, calcium score, myocardial blood flow

## Abstract

**Background:**

Imaging-based measures of atherosclerosis such as coronary artery calcium score (CACS) and coronary flow reserve (CFR) as well as carotid atherosclerotic plaque burden (cPB) are predictors of cardiovascular events in the general population. The objective of this study was to correlate CACS, cPB, myocardial blood flow (MBF), and CFR in patients with end-stage renal disease (ESRD).

**Methods and results:**

39 patients (mean age 53 ± 12 years) with ESRD prior to kidney transplantation were enrolled. MBF and CFR were quantified at baseline and under hyperemia by ^13^N-NH_3_-PET/CT. CACS was calculated from low-dose CT scans acquired for PET attenuation correction. cPB was assessed by 3D ultrasound. Uni- and multivariate regression analyses between these and clinical parameters were performed. Median follow-up time for clinical events was 4.4 years. Kaplan–Meier survival estimates with log-rank test were performed with regards to cardiovascular (CV) events and death of any cause. CACS and cPB were associated in ESRD patients (*r *= 0.48; *p *≤ 0.01). While cPB correlated with age (*r *= 0.43; *p* < 0.01), CACS did not. MBF_stress_ was negatively associated with age (*r *= 0.44; *p *< 0.01) and time on dialysis (*r *= 0.42; *p *< 0.01). There were negative correlations between MBF_stress_ and CACS (*r *= − 0.62; *p *< 0.001) and between MBF_stress_ and cPB (*r *= − 0.43; *p *< 0.01). Age and CACS were the strongest predictors for MBF_stress_. CFR was impaired (< 2.0) in eight patients who also presented with higher cPB and higher CACS compared to those with a CFR > 2.0 (*p *= 0.06 and *p *= 0.4). In contrast to MBF_stress_, there was neither a significant correlation between CFR and CACS (*r *= − 0.2; *p *= 0.91) nor between CFR and cPB (*r *= − 0.1; *p *= 0.55). CV event-free survival was associated with reduced CFR and MBF_stress_ (*p *= 0.001 and *p *< 0.001) but not with cPB or CACS.

**Conclusions:**

CACS, cPB, and MBF_stress_ are associated in patients with ESRD. Atherosclerosis is earlier detected by MBF_stress_ than by CFR. CV event-free survival is associated with impaired CFR and MBF_stress_.

**Electronic supplementary material:**

The online version of this article (10.1007/s12350-020-02080-w) contains supplementary material, which is available to authorized users.

## Introduction

Chronic kidney disease (CKD) is a major risk factor for the development of atherosclerosis and coronary artery disease (CAD) and patients with CKD are at increased risk for myocardial infarction, heart failure, and cardiac death.^1^,^2^ The pathophysiological interaction between the kidneys and the heart is termed the cardiorenal syndrome (CRS), with especially high risk for cardiovascular events in end-stage renal disease (ESRD) patients.^3^ Besides CAD of the large coronary arteries which often remains asymptomatic, microvascular disease, i.e., coronary microvascular dysfunction, is a frequent finding in this high-risk group.^4^

However, questions remain of how to assess cardiovascular risk in individual ESRD patients and how to identify patients who may benefit from further testing or a coronary intervention prior to transplantation.^5^,^6^ In suspected or known CAD the role of non-invasive myocardial perfusion imaging (MPI) in preselecting patients for coronary angiography is well established. Single photon emission computed tomography (SPECT) is a widely used imaging method with good diagnostic accuracy for the detection of relevant epicardial coronary artery disease in CKD patients.^7^ However, microvascular alterations may be missed by SPECT. Thus, quantitative myocardial perfusion positron emission computed tomography (PET) is the method of choice for quantitative MPI, as it additionally quantifies absolute myocardial blood flow (MBF) and coronary flow reserve (CFR) – increasing diagnostic accuracy and adding prognostic information.^4^,^8^,^9^ Recently, global CFR was proven to be an independent factor for risk stratification for all-cause and cardiovascular mortality in ESRD.^4^ Another imaging-derived parameter of atherosclerosis is the coronary artery calcium score (CACS), which also showed independent prognostic value in suspected CAD.^11^^–^13

Atherosclerosis – as a systemic disease – also causes carotid artery stenosis and peripheral artery disease.^10^ Apart from the calculation of intima to media thickness,^10^,^14^ carotid ultrasound enables the quantitation of carotid artery atherosclerotic plaque burden (cPB).^15^ cPB was the strongest predictor of coronary artery calcium score (CACS) in asymptomatic, otherwise healthy individuals.^16^ In the general population, cPB and CACS can serve as imaging biomarkers of future cardiovascular events in asymptomatic adults.^16^ However, the relation between myocardial perfusion, CACS, and cPB has not yet been studied in ESRD patients. Therefore, the aim of this study was to correlate the results from myocardial perfusion PET with CACS and cPB in a cohort of ESRD patients prior to kidney transplantation and to evaluate if CFR or absolute MBF are better indicators for coronary artery pathology.

## Methods

### Study Population

39 patients (14 female; 36%), mean age 53 ± 12 years (range 26-69) with ESRD on dialysis [12 peritoneal dialysis (PD); 27 hemodialysis (HD)], were prospectively enrolled in the study prior to kidney transplantation. Exclusion criteria were renal failure in a pre-dialysis state, prior kidney transplantation, or known CAD in patients individual history. All patients were asymptomatic at the time of imaging. Detailed patient characteristics are provided in Table 1.

**Table 1:** Patients characteristics

variable	all patients (N = 39)	PD (N = 12)	HD (N = 27)	*p* value
Demographics				
Age (years)	53 (26–69)	52 (26–69)	54 (27–69)	0.59
Women	14 (36%)	6 (50%)	8 (30%)	0.29
Duration of dialysis				
< 1 year	14 (36%)	6 (50%)	8 (30%)	0.65
1–3 years	15 (38%)	3 (25%)	12 (44%)	0.41
> 3 years	10 (26%)	3 (25%)	7 (26%)	0.28
Cardiovascular risk factors				
Hypertension	39 (100%)	12 (100%)	27 (100%)	
Diabetes	9 (23%)	4 (33%)	5 (18.5%)	0.24
Hypercholesterolemia	18 (46%)	8 (67%)	10 (37%)	0.11
Family history of CAD	6 (15%)	1 (8%)	5 (18.5%)	0.44
BMI (kg/m^2^)	27.2 (18.5–37.1)	26.8 (19.3–32.4)	27.4 (18.5–37.1)	0.69
Medication				
Calcium channel blockers	24 (62%)	6 (50%)	18 (67%)	0.32
ß-blockers	30 (77%)	10 (83%)	20 (74%)	0.53
Statins	11 (28%)	5 (42%)	6 (22%)	0.21
ACE inhibitors	7 (18%)	3 (25%)	4 (15%)	0.44
Angiotensin receptor blockers	18 (46%)	6 (50%)	12 (44%)	0.75
Diuretics	23 (59%)	7 (58%)	16 (59%)	0.96
Serum laboratories				
Total cholesterol	193 (128–370)	240 (164–370)	179 (128–236)	**0.01**
LDL	117 (64–246)	159 (92–246)	103 (64–133)	**0.01**
Triglycerides	193 (85–224)	212 (122–376)	187 (85–424)	0.59
Hb	11.2 (8.3–13.7)	11.9 (10.0–13.7)	10.9 (8.3–13.4)	**0.02**
Uric acid	5.9 (1.9–10.5)	5.9 (3.0–9.3)	6.0 (1.9–10.5)	0.81
Imaging parameters				
LVEF rest	54 (29–73)	55 (42–69)	54 (29–73)	0.86
LVEF stress	53 (33–70)	54 (42–69)	52 (33–64)	0.58
Ischemia (% myocardium)	0.7 (0–9)	1.2 (0–9)	0.4 (0–6)	0.36
Scar (% myocardium)	0.1 (0–3)	0 (0)	0.1 (0-3)	0.51
CFR	2.5 (1.4–4.7)	2.6 (1.5–4.7)	2.5 (1.4–3.9)	0.87
MBF stress (ml/g per minute)	2.8 (1.6–4.6)	2.9 (1.8–4.6)	2.7 (1.6–3.9)	0.44
MBF rest (ml/g per minute)	1.2 (0.7–2.2)	1.3 (0.7–2.2)	1.1 (0.7–1.6)	0.35
cPB	277 (0–801)	180 (0–396)	320 (0–801)	**0.04**
CACS	440 (0–3957)	340 (0–2243)	484 (0–3957)	0.65

*CAD*, coronary artery disease; *BMI*, body mass index; *ACE*, angiotensin converting enzyme; *LDL*, low density lipoprotein; *Hb*, hemoglobin; *LVEF*, left ventricular ejection fraction; *CFR*, coronary flow reserve; *MBF*; myocardial blood flow; *cPB*, carotid plaque burden; *CACS*; coronary artery calcium score

### PET Acquisition

A 128-slice PET/CT scanner (Biograph mCT, Siemens Healthcare) was used for PET imaging. Patients fasted for > 4 hour and refrained from caffeine or theophylline for 24 hour before imaging. A low-dose CT scan of the heart (100 kV, 60 mA with CARE Dose, 0.5 second per rotation, Pitch 1.2; 3 mm reconstructed slice thickness) for attenuation correction of PET data was acquired during shallow breathing. For the rest study ^13^N-NH_3_ (352 ± 38 MBq) was given in an intravenous bolus. List-mode 3-dimensional PET images were acquired for 14 minutes. Infusion of adenosine was started through a separate intravenous line (0.14 µg/kg, 6 minutes) and a second dose of ^13^N-NH_3_ (354 ± 39 MBq) was infused at the half time of adenosine infusion, followed by a 14 minutes. list-mode acquisition. Quality control of PET and CT for attenuation correction was performed. List-mode data were resampled to attenuation-corrected static, electrocardiographically gated, and dynamic images (17 frames; 12 × 10 seconds; 3 × 20 seconds and 2 × 30 seconds). Emission data were corrected for randoms, dead time, scatter, and attenuation. All images were reconstructed iteratively by a 3D attenuation-weighted ordered-subsets expectation maximization iterative reconstruction algorithm and time-of-flight (OSEM 3D; 2i21s, Gaussian smoothing at 8 mm full-width at half-maximum; zoom of 1).

PET data were analyzed using commercially available software (syngo.PET Myocardial Blood Flow and MI Cardiology; syngo.via; Siemens Healthcare, Germany).

### Analysis of Regional Perfusion

Static perfusion images were reangulated to short, horizontal, and vertical long axis slices for visual analysis. Each of the 17 myocardial segments was scored on a scale of 0-4 (normal, mildly, moderately, severely decreased, or absent perfusion) by two independent and experienced readers blinded to other data. Summed stress score (SSS), summed rest score (SRS), and summed difference score (SDS) were calculated. Abnormal perfusion was defined as a SSS ≥ 4. Significant ischemia was defined as a SDS ≥ 3. Coronary angiography was performed in these selected cases.

### Quantification of MBF, CFR, and Left Ventricular Function

Absolute MBF (ml·g·min) was calculated from the dynamic stress and rest scans corrected for motion. Automated factor analysis was used to generate arterial input function and myocardial tissue time-activity curves. Hyperemic MBF (MBF_stress_) was corrected for myocardial ^13^N-NH_3_ activity (from the rest study). MBF_stress_ and resting MBF (MBF_rest_) were calculated by fitting the time-activity curves to a 2-compartment kinetic model as described elsewhere.^17^ MBF_rest_ was normalized for cardiac work by dividing by the rate–pressure product and multiplying the result by 10.000.^18^ CFR was determined as the ratio of MBF_stress_ to corrected MBF_rest_. Stress and resting left ventricular ejection fraction (LVEF_stress_ and LVEF_rest_) were calculated from the ECG-triggered PET data using the Corridor 4DM software (Invia, Ann Arbor, USA).

### Coronary Artery Calcium Scoring

CACS assessment was performed using the low-dose, non-contrast multidetector CT scans for attenuation correction without prospective electrocardiography-gated acquisition. CACS was calculated by the Agatston method accounting for the area of coronary artery calcification in CT (> 130 Hounsfield units, preset value by the calcium scoring software, Siemens Healthcare, Germany) and expressed as Agatston score^19^ into the following categories: ≤ 100; 101-400, 401-1000 and > 1000.^20^

### 3D Carotid Ultrasound

The Philips iU22 ultrasound system (Philips Healthcare, Bothell, Washington) with a dedicated 3D transducer was used. All scans were performed by physicians with advanced experience in head and neck ultrasound imaging. The protocol included 3D imaging of the left and right common carotid artery and its bifurcation as far as assessable. All 3D-ultrasound recordings were analyzed using dedicated software for semiautomatic plaque quantification (Philips QLAB-VPQ). Carotid plaque burden (cPB) was expressed in mm^3^.^16^ cPB patients were stratified in the following tertiles: ≤ 154 mm^3^, 155-344 mm^3^, and ≥ 345 mm^3^ (13 patients each).

### Follow-Up

Clinical follow-up was performed every 6 months. This included clinical patient interview, laboratory data, and recording of clinical events. End-points were major cardiovascular (CV) events and death from any cause.

### Statistical Analysis

Analysis of discrete variables was performed using the Chi squared test. Continuous variables were compared either by a two-sided Student’s *t* test for unpaired samples or by one-way analysis of variance (ANOVA) with a Bonferroni/Tamhane correction. Univariate and multivariate logistic regression analyses were performed to test for associations between continuous variables. Pearson’s correlation or Spearman-Rho tests were used for the comparison of parametric normally or not normally distributed variables. Variables significantly correlated in the univariate analysis were included in a multivariate stepwise regression model. Two-sided *p* values < 0.05 were considered significant. Kaplan–Meier survival curves with log-rank test were performed for the analysis of patients survival. Analyses were performed with SPSS software package (IBM SPSS Statistics, Version 22.0. Armonk, NY).

All procedures performed were in accordance with the ethical standards of the institutional and research committee and with the principles of the 1964 Declaration of Helsinki and its later amendments. The study was approved by the institutional review board of the Medical Association of Westfalen-Lippe and the Faculty of Medicine, University of Münster. Informed consent was given by all patients.

## Results

Detailed characteristics of the patient cohort are provided in Table 1.

### CACS and cPB

**Figure. 1:**
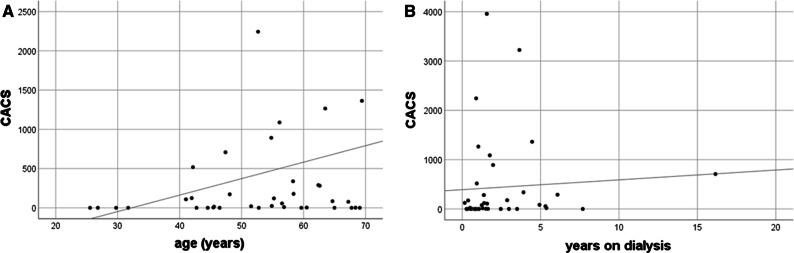
Scatterplot diagrams of CACS in correlation to age (**A**) and time on dialysis (**B**)

22 (56%) individuals had a CACS < 100, eight patients (21%) had a CACS between 101 and 400 and nine patients (23%) had a CACS > 401 (six of which > 1000). CACS was not correlated with age (*r* = 0.27; *p* = 0.97; Figure 1A) or with the time on dialysis (*r* = 0.28; *p* = 0.08; Figure 1B). CACS did not differ between the PD and HD subgroups (Table 1).

**Figure. 2:**
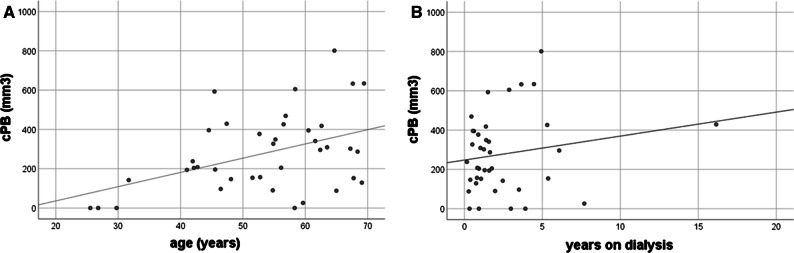
Scatterplot diagrams of cPB in correlation to age (**A**) and time on dialysis (**B**)

cPB correlated with age (*r* = 0.43; *p* = 0.007; Figure. 2A) but not with the time on dialysis (*r* = 0.18; *p* = 0.28; Figure. 2B). cPB was significantly higher in the HD as compared to the PD subgroup (Table 1). cPB showed a moderate association with CACS (*r* = 0.48; *p* = 0.002).

### MBF, CFR, and Left Ventricular Function

**Figure. 3:**
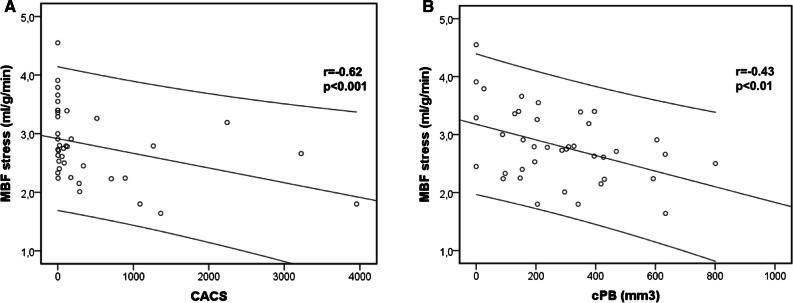
Correlation between absolute MBF_stress_ and CACS (**A**) and cPB (**B**), respectively

MBF_rest_ ranged from 0.7 to 2.2 ml·g·min and MBF_stress_ ranged from 1.6 - 4.6 ml·g·min. Individuals with MBF_stress_ < 2 ml·g·min (N = 3) presented with higher cPB (393 ± 219 vs 268 ± 197 mm^3^) and higher CACS (2136 ± 1583 vs. 299 ± 671) compared to those with MBF_stress_ > 2 ml/g/min. Mean MBF_stress_ did not differ between patients on PD or HD (Table 1). There was a negative correlation between MBF_stress_ and CACS (*r* = − 0.62; *p* < 0.0001; Figure. 3A) and between MBF_stress_ and cPB (*r* = − 0.43; *p* = 0.007; Figure. 3B). MBF_stress_ impaired with increasing CACS and cPB, respectively. In contrast, CFR remained stable with increasing CACS and cPB (Table 2), since MBF_rest_ decreased. Moreover, there was a moderate correlation between MBF_stress_ and LVEF_rest_ (*r* = 0.32; *p* = 0.05) but there was no correlation between CFR and LVEF_rest_ (*r* = 0.14; *p* = 0.94).

**Table 2:** CFR, MBF (rest, stress), and Hb dependent on CACS and cPB

	Gender		Age	MBF_rest_ (ml/g/min)		MBF_stress_ (ml/g/min)		CFR global		Hemoglobin	
	Male (N)	Female (N)	Years	Mean	SD	Mean	SD	Mean	SD	Mean	SD
CACS categories											
0–100	9	13	51.5 ± 13.9	1.3	0.3	3.0	0.6	2.5	0.7	11.2	1.1
101–400	7	1	53.5 ± 8.7	1.1	0.2	2.6	0.5	2.5	0.8	10.8	1.8
401–1000	3	0	48.1 ± 6.4	1.0	0.2	2.6	0.6	2.7	0.5	11.9	1.5
> 1000	6	0	61.8 ± 6.5	1.0	0.3	2.3	0.6	2.6	1.1	11.7	1.7
cPB tertiles											
First	5	8	48.8 ± 15.7	1.2	0.3	3.1	0.7	2.7	0.7	10.9	1.1
Second	8	5	53.9 ± 10.2	1.2	0.3	2.7	0.5	2.3	0.5	11.3	1.4
Third	12	1	57.1 ± 8.0	1.1	0.4	2.6	0.5	2.7	0.9	11.4	1.7

*CFR*, coronary flow reserve; *MBF*, myocardial blood flow; *cPB*, carotid plaque burden; *CACS*, coronary artery calcium score; *SD*, standard deviation

**Figure. 4:**
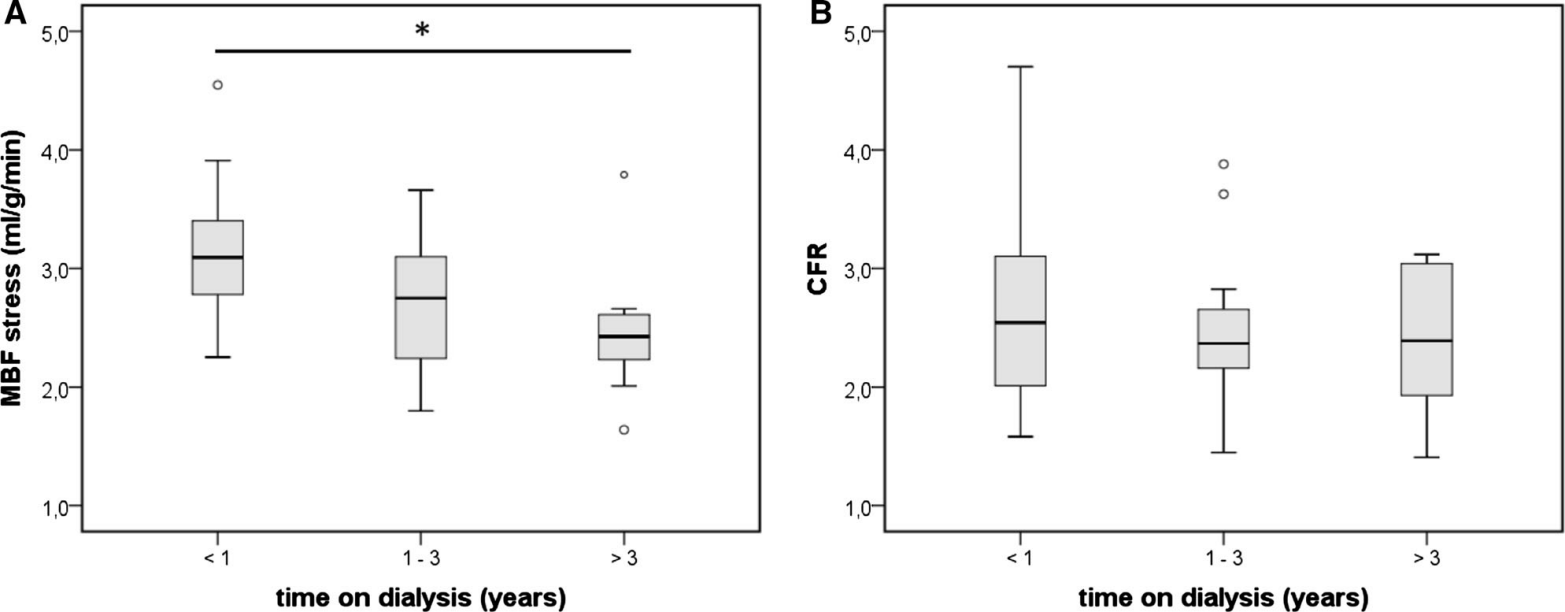
MBF_stress_ (**A**) and CFR (**B**) dependent from the time on dialysis. * indicates significant differences (*p *< 0.05)

MBF_stress_ was negatively associated with age (*r* = 0.44; *p* < 0.01) and the time on dialysis (*r* = 0.42; *p* < 0.01). MBF_stress_ decreased from 3.16 ± 0.58 ml·g·min. in patients < 1 year on dialysis to 2.46 ± 0.56 ml·g·min. in patients > 3 years on dialysis (*p* < 0.05; Figure 4A). In contrast, CFR did not change (*p* = 1.0; Figure. 4B).

CFR ranged from 1.4 to 4.7 in the total cohort. There were also no significant differences between patients on PD or HD (Table 1). CFR was impaired (< 2.0) in eight patients (20%; *p* = 0.47). These individuals also presented with higher cPB (394 ± 169 vs 247 ± 197 mm^3^) and a higher CACS (679 ± 1119 vs 379 ± 833) compared to those with a CFR > 2.0 (*p* = 0.06 and *p* = 0.4, respectively). There was neither a significant correlation between CFR and CACS (*r* = − 0.2; *p* = 0.91) nor between CFR and cPB (*r* = − 0.1; *p* = 0.55). There were inverse correlations between MBF_rest_ and hemoglobin (*r* = − 0.39; *p* = 0.015) and between CFR and hemoglobin (*r* = 0.32; *p* = 0.048).

Significant correlations are summarized in Table 3. In a stepwise multivariate regression analysis hemoglobin proved as strongest predictor for MBF_rest_. Age and CACS were the strongest predictors for MBF_stress_ whereas only age was predictive for cPB.

**Table 3:** Summary of significant correlations

Parameter	Univariate	
	cc	*p* value
CFR_global_		
***Hb***	0.318^b^	0.048
MBF_rest_		
*Hb*	− 0.386^b^	0.015
*CACS*	− 0.424^b^	0.007
MBF_stress_		
**Age**	− 0.439^a^	0.005
*cPB*	− 0.425^a^	0.007
***CACS***	− 0.619^b^	<0.0001
Time from first dialysis	− 0.419^b^	0.008
Summed years of dialysis	− 0.413^b^	0.009
cPB		
**Age**	− 0.425^a^	0.007
*CACS*	0.476^b^	0.002
CACS		
MB_stress_	− 0.619^b^	<0.0001
MB_rest_	− 0.424^b^	0.007
*cPB*	0.476^b^	0.002

Variables independently correlated in a multivariate analysis are presented in bold type

*Hb*, hemoglobin; *CACS*, coronary artery calcium score; *cPB*, carotid plaque burden; *MBF*, myocardial blood flow; *cc*, correlation coefficient

^a^Pearson’s *r*

^b^Spearman’s rho

### Ischemia

Reversible stress perfusion defects were observed in four patients (10%). In these cases ischemia ranged from 4 to 9%. Global CFR was ≥ 2.0 in all cases and MBF_stress_ was impaired (< 2.0 ml·g·min) in only one case. None of the patients had significant coronary artery stenoses > 50% in subsequent coronary angiography.

### Predictive Value

10 patients (26%; two men, eight women) presented with CACS < 100 and cPB < 154 mm^3^ (= lower tertile limit), whereas 29 patients (74%; 23 men, six women) presented with either CACS > 100 or cPB > 154 mm^3^ or both. None of the patients with low CACS and cPB presented with impaired CFR or MBF_stress_. Among the patients with high CACS and/or cPB only eight patients (27.5%) showed impaired CFR and three (10%) impaired MBF_stress_. Patients with low CACS and cPB were older by trend (55.3 ± 8.8 vs 47.3 ± 17.7 years; *p* = 0.2) but there were no differences regarding the time on dialysis (2.4 ± 3.1 vs 2.5 ± 2.4 years; *p* = 0.91).

**Figure. 5:**
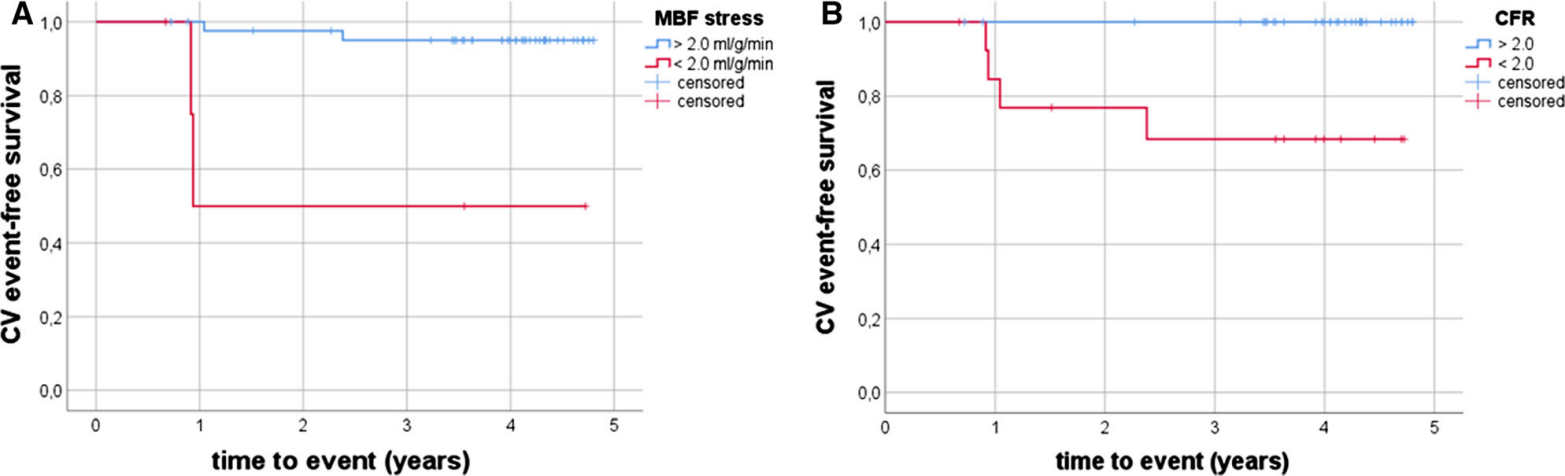
Kaplan-Meier plots showing CV event-free survival in order to MBF_stress_ (**A**) and CFR (**B**)

Median follow-up time was 4.4 years (mean 3.6 ± 1.1 years). In the follow-up, there were four major cardiovascular events (MACE): 2 myocardial infarctions, 1 stroke, and 1 pulmonary embolism. There were six deaths, all by non-cardiovascular causes. CV event-free survival was associated by reduced CFR and MBF_stress_ (*p* = 0.001 and *p* < 0.001) but not with cPB or CACS (Figure. 5). On the other hand overall survival could not be predicted by any of these variables (all *p* > 0.25).

## Discussion

We investigated the association between quantitative MBF, CACS and cPB in patients with ESRD assigned for kidney transplantation evaluation. Our main findings were: MBF_stress_ but not CFR was significantly correlated with CACS and cPB. The strongest predictors for MBF_stress_ were CACS and age, whereas cPB was best predicted by age. On the other hand the strongest predictor for CACS was MBF_stress_. Particularly the combination of low CACS and low cPB resulted in a high negative predictive value for preserved MBF and CFR.

The BioImage Study investigated the relationship of several (imaging) biomarkers and cardiovascular outcome in a large population of asymptomatic adults without known cardiovascular or renal disease.^16^ Here, a distinct correlation between CACS and cPB was described. In another study cPB was shown to be predictive of mortality and to occur more frequently in patients on HD than on PD,^21^ the latter also observed in our study. In line with the BioImage Study, we found a significant correlation between CACS and cPB also in patients with ESRD. In a multivariate regression analysis the strongest predictors for CACS and cPB were MBF_stress_ and age. Thus, the results of our study are in line with the current understanding of atherosclerosis as a systemic and diffuse disease. Interestingly, cPB was only moderately associated with and not predictive of MBF_stress_ in the multivariate analysis. Instead, CACS turned out to be predictive for MBF_stress_. This is not surprising since CACS reflects the extent of coronary artery atherosclerosis which directly influences myocardial perfusion and the degree of atherosclerosis in the carotid arteries does not necessarily preclude coronary atherosclerosis with a similar extent. Our findings indicate that none of the imaging parameters is able to predict the others with sufficient accuracy in patients with ESRD. Importantly, CACS is not limited to intimal calcification but also superposed by smooth muscle cell mediated, metabolic media calcification in patients with ESRD.^22^ This also could explain the only moderate correlation between CACS and cPB, since cPB is rather a marker of ‘classical’ atherosclerosis resulting from subintimal plaque development. Due to this different pathophysiology, the clinical value of CACS in CKD patients still remains controversial.^13^ Interestingly, CACS did not correlate with age. This finding may be explained by the different pathophysiology of media calcification but also (at least partly) by the distinctly younger age of patients studied here as compared with a classical CAD population.

Another factor associated with MBF_stress_ was the time on dialysis. This finding supports the assumption that the duration of renal replacement therapy has a strong impact on myocardial perfusion, mainly due to rarefication of small intramyocardial vessels.^23^–^25^ We found impaired CFR in 20% of our patients, which is less than reported in other studies^33^ and most probably due to the relatively young study cohort. Prior investigations have suggested that CKD is associated with abnormal coronary vasodilator function,^26^ which results from multiple mechanisms including decreased capillary density,^27^ vascular remodeling,^28^ and endothelial dysfunction.^29^,^30^ Early stage microvascular disease may be the reason for impaired MBF_stress_ in our patient cohort. This can be assumed since global CFR was still preserved and significant epicardial CAD could be excluded by angiography in selected cases with mild to moderate regional ischemia. Thus, we interpret the data in that way that regional ischemia is most probably related to regional microvascular dysfunction, since technical image artifacts could be excluded. This can be missed by the assessment of *global* CFR.

Although preliminary results based on a rather small patient cohort should always be interpreted with care, our results indicate that the combination of low cPB and low CACS may have a high negative predictive value with regards to the exclusion of CAD. On the other hand the positive predictive value of CACS and cPB for CFR and MBF was low. Thus, it would be worth to evaluate in a larger study if the assessment of cPB and CACS may replace MPI in the evaluation prior to kidney transplantation listing in cases of normal findings. But on the other hand in this context importantly, our results indicate that cardiovascular event-free survival may be not predicted by cPB or CACS but only by CFR and MBF_stress_. These findings are in line with previous study results in distinctly larger ESRD patient cohorts already showing the prognostic value of CFR in this particular patient group, although in these studies a lower cutoff value for CFR than in our study was used.^4^,^31^

**Figure. 6:**
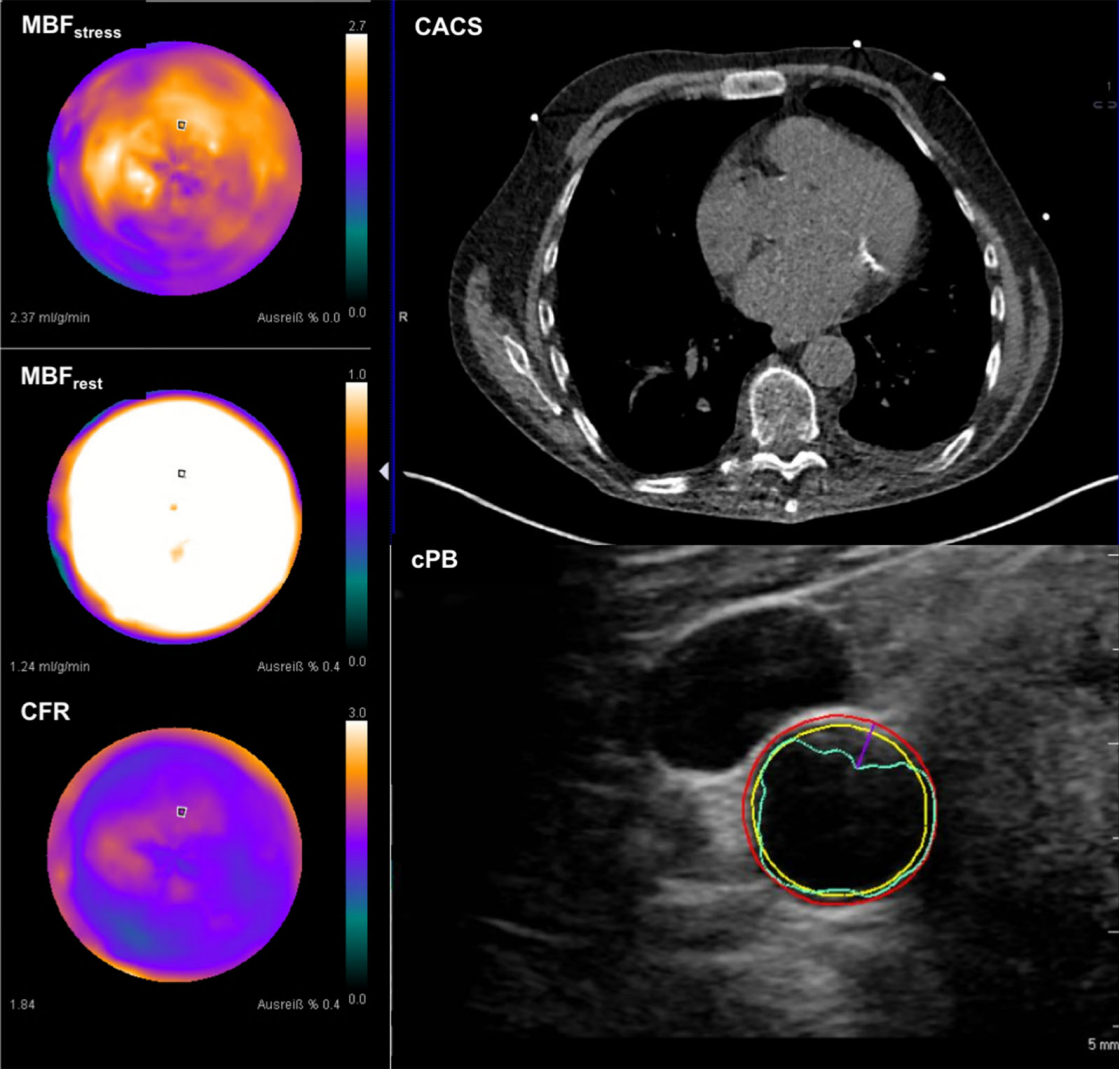
Example of a patient with globally impaired CFR (1.7) mostly due to an elevated MBF_rest_. (1.4 ml·g·min.). MBF_stress_ was 2.0 ml·g·min., moderate CACS- and cPB values (Agatston score: 289; cPB: 296 mm^3^)

Notably striking was the observation that CFR was independently associated with the current Hb value and was neither correlated with CACS or cPB nor with age or the time on dialysis. Increased MBF_rest_ has been already observed in mild CKD and was correlated with the degree of GFR.^32^ Anemia induces a compensatory increase in MBF_rest_ via increased nitric oxide release^33^ which naturally also affects CFR. Although in former investigations impaired CFR was highly prevalent in patients with CKD (4, 35; example Figure 6), our results suggest that MBF_stress_ may be a better parameter for the early assessment of microvascular dysfunction. Variability of MBF_rest_ in the individual person is widely known and is probably related to differences in myocardial work load.^35^ Although corrected for cardiac work increased MBF_rest_ due to other factors as metabolic oxygen demand or an enhanced activation of the sympathetic nervous and renin–angiotensin–aldosterone system^34^ which is chronically activated in patients with ESRD cannot be ruled out. Therefore, when interpreting CFR, changes in MBF_rest_ due to alterations in metabolic demand need to be considered.^36^–^38^ Altogether, the results of our study raise the question if absolute MBF_stress_ may be an earlier and more robust marker for CAD in patients with ESRD than CFR. This hypothesis is additionally supported by the finding that only MBF_stress_ was significantly correlated with LVEF_(rest)_ but not CFR. In former studies absolute MBF_stress_ already shown to be more accurate than CFR in detecting CAD in non-CKD patients.^35^,^37^ Interestingly, MBF_rest_ was higher in patients without or minimal CACS and low cPB, a finding which is most probably caused by the higher frequency of females in these groups. Most studies report of higher MBF_rest_ in females, although the mechanisms of this phenomenon remain uncertain.^38^

### Study Limitations

The small size of the patient cohort is certainly a limitation of our study. Furthermore, the study population was young and time on dialysis was relatively short. These factors may explain the relatively low number of pathological findings. Another limitation is the use of a non-ECG-triggered, low-dose CT for CACS although CACS on that basis is feasible.^39^,^40^ Thus, a dedicated state-of-the-art CACS-CT was not performed additionally in order to avoid futile additional radiation exposure.

## New Knowledge Gained

Our results indicate that MBF_stress_ is a marker of subclinical atherosclerosis in patients with ESRD, whereas CFR may miss early stages of vascular dysfunction in this patient cohort. Nevertheless, impaired CFR and MBF_stress_ are predictors for CV event-free survival whereas cPB and CACS may be not.

## Conclusions

Although CACS and cPB as parameters of coronary and systemic atherosclerosis are associated in patients with ESRD, only absolute MBF_stress_ but not CFR correlates with CACS and cPB. Furthermore, MBF_stress_ impairs with increasing time on renal replacement therapy. MBF_rest_ and CFR are regularly influenced by actual hemoglobin values. Thus, MBF_stress_ may serve as a robust parameter for the detection of microvascular disease in patients with ESRD than CFR.

Overall, our results indicate that quantitative MPI cannot be replaced by the assessment of CACS or cPB only. Nevertheless, there may be a good negative predictive value. CV event-free survival is associated with impaired CFR and MBF_stress_ only.

## Electronic supplementary material

Below is the link to the electronic supplementary material.

Supplementary material 1 (PPTX 5706 kb)

## Abbreviations

CKD: Chronic kidney disease
CAD: Coronary artery disease
ESRD: End-stage renal disease
MPI: Myocardial perfusion imaging
MBF: Myocardial blood flow
CFR: Coronary flow reserve
cPB: Carotid artery atherosclerotic plaque burden
CACS: Coronary artery calcium score
PD: Peritoneal dialysis
HD: Hemodialysis
