# Supplementary material for: Coronary artery calcium burden, carotid atherosclerotic plaque burden, and myocardial blood flow in patients with end-stage renal disease: A non-invasive imaging study combining PET/CT and 3D ultrasound
Source: J Nucl Cardiol. 2020 Mar 5;28(6):2660–70. doi: 10.1007/s12350-020-02080-w (PMC8709813; doi:10.1007/s12350-020-02080-w)
Supplement: Supplementary file 1 — Supplementary material 1 (PPTX 5706 kb) [file 12350_2020_2080_MOESM1_ESM.pptx]

## Slide 1
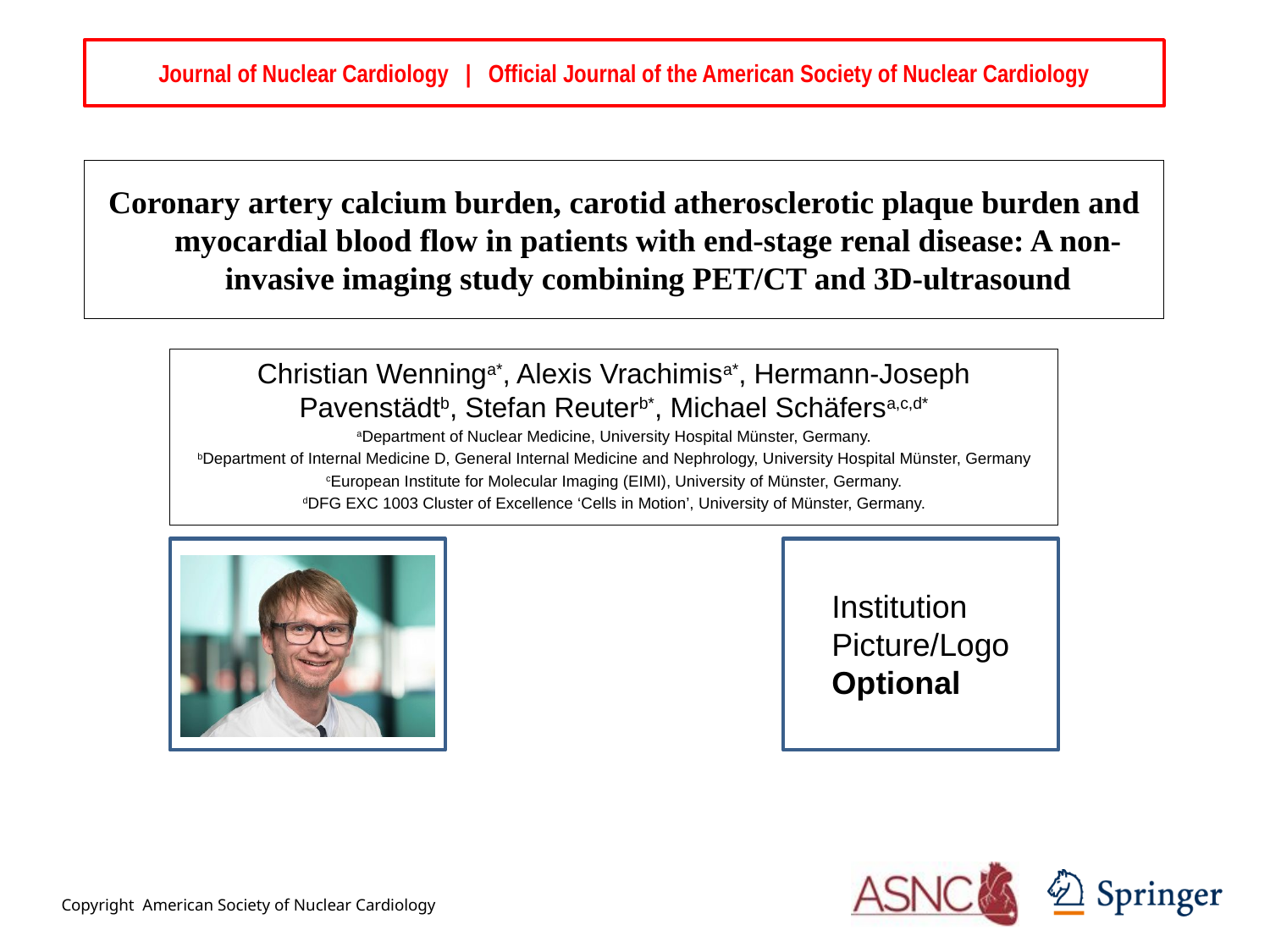

Journal of Nuclear Cardiology | Official Journal of the American Society of Nuclear Cardiology
# Coronary artery calcium burden, carotid atherosclerotic plaque burden and myocardial blood flow in patients with end-stage renal disease: A non-invasive imaging study combining PET/CT and 3D-ultrasound
Christian Wenninga*, Alexis Vrachimisa*, Hermann-Joseph Pavenstädtb, Stefan Reuterb*, Michael Schäfersa,c,d*
aDepartment of Nuclear Medicine, University Hospital Münster, Germany.
bDepartment of Internal Medicine D, General Internal Medicine and Nephrology, University Hospital Münster, Germany
cEuropean Institute for Molecular Imaging (EIMI), University of Münster, Germany.
dDFG EXC 1003 Cluster of Excellence ‘Cells in Motion’, University of Münster, Germany.
Head shot of author
required
Institution
Picture/Logo
Optional
Copyright American Society of Nuclear Cardiology

## Slide 2
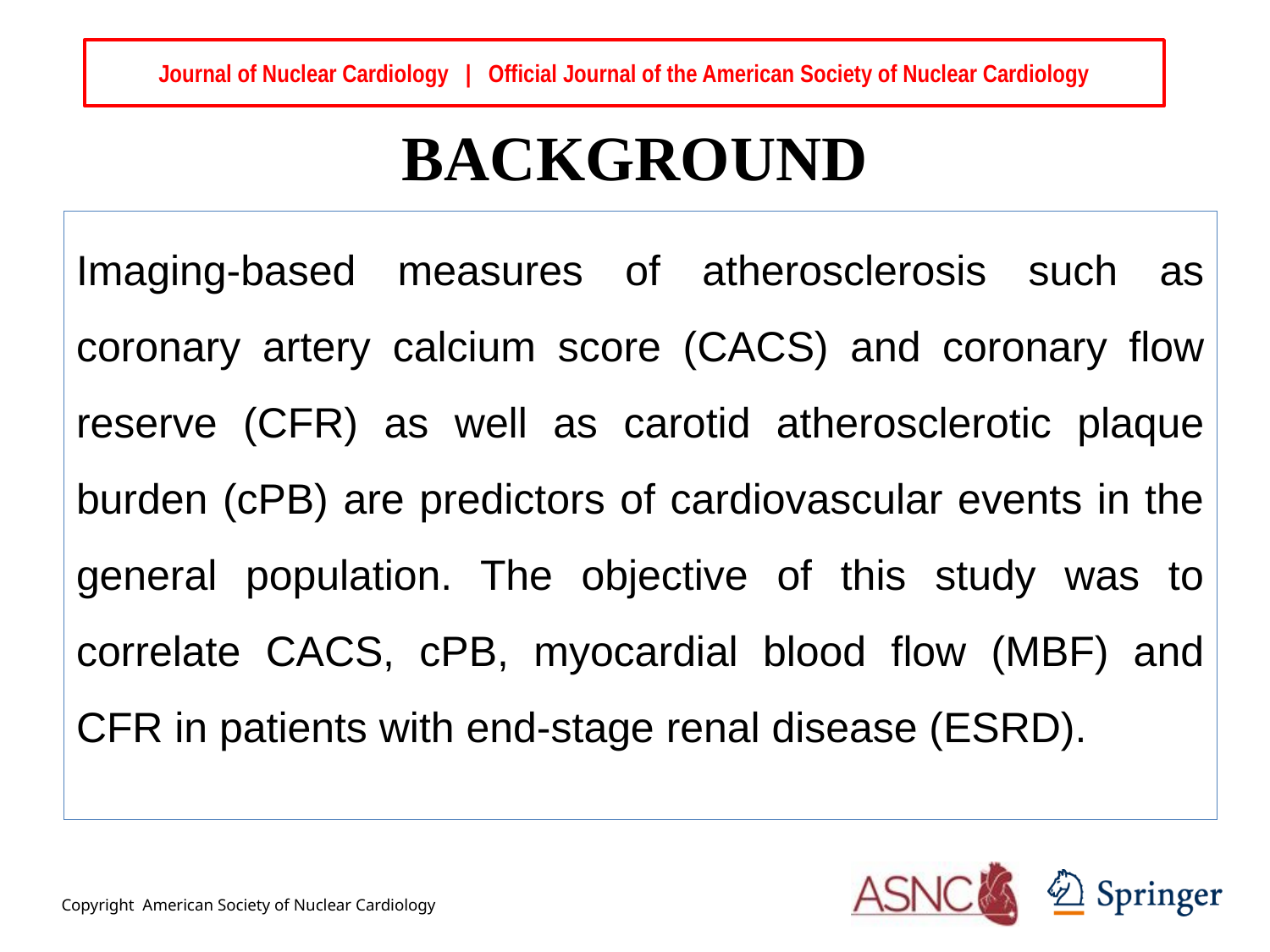

Journal of Nuclear Cardiology | Official Journal of the American Society of Nuclear Cardiology
# BACKGROUND
Imaging-based measures of atherosclerosis such as coronary artery calcium score (CACS) and coronary flow reserve (CFR) as well as carotid atherosclerotic plaque burden (cPB) are predictors of cardiovascular events in the general population. The objective of this study was to correlate CACS, cPB, myocardial blood flow (MBF) and CFR in patients with end-stage renal disease (ESRD).
Copyright American Society of Nuclear Cardiology

## Slide 3
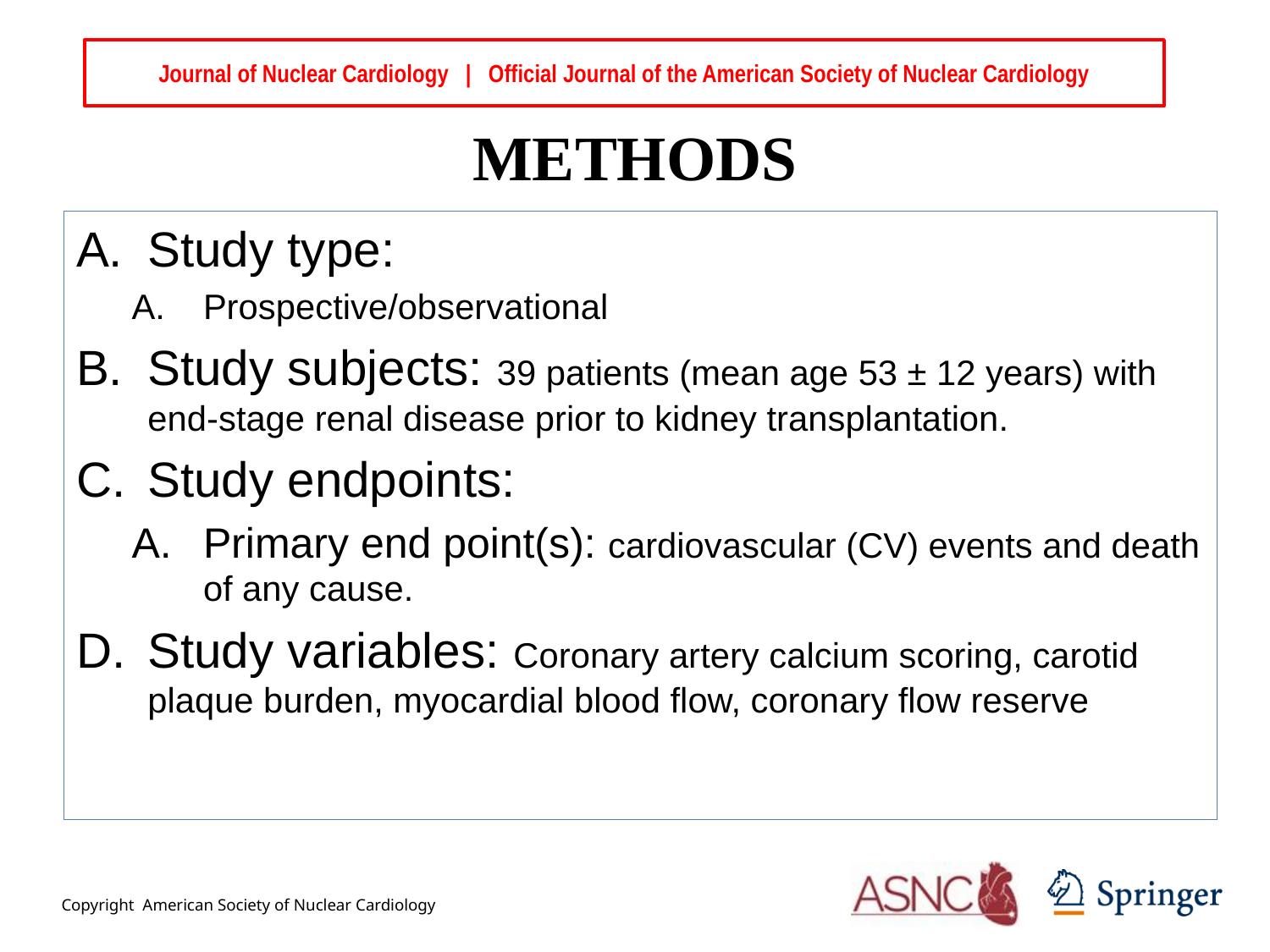

Journal of Nuclear Cardiology | Official Journal of the American Society of Nuclear Cardiology
# METHODS
Study type:
Prospective/observational
Study subjects: 39 patients (mean age 53 ± 12 years) with end-stage renal disease prior to kidney transplantation.
Study endpoints:
Primary end point(s): cardiovascular (CV) events and death of any cause.
Study variables: Coronary artery calcium scoring, carotid plaque burden, myocardial blood flow, coronary flow reserve
Copyright American Society of Nuclear Cardiology

## Slide 4
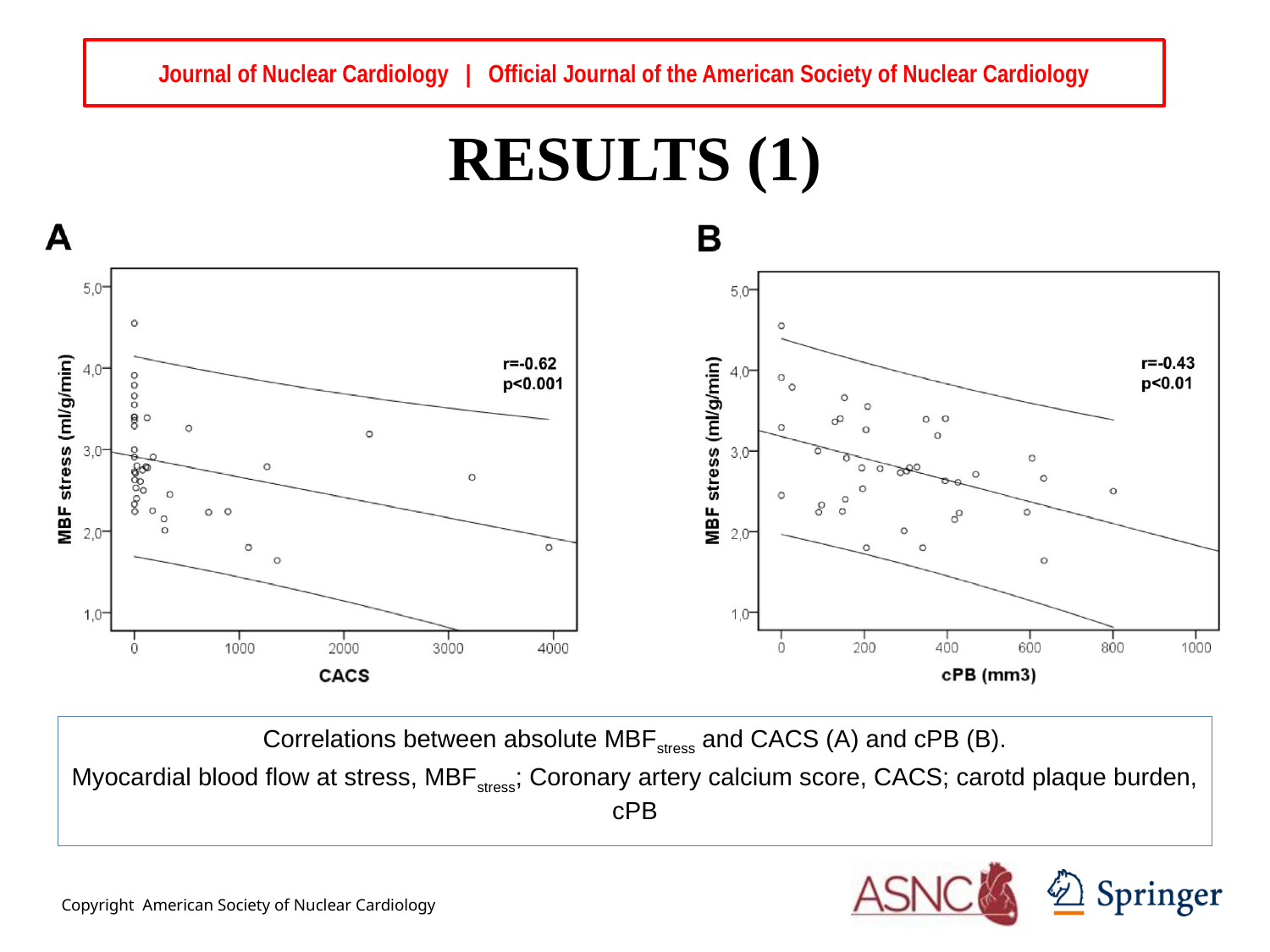

Journal of Nuclear Cardiology | Official Journal of the American Society of Nuclear Cardiology
# RESULTS (1)
Correlations between absolute MBFstress and CACS (A) and cPB (B).
Myocardial blood flow at stress, MBFstress; Coronary artery calcium score, CACS; carotd plaque burden, cPB
Copyright American Society of Nuclear Cardiology

## Slide 5
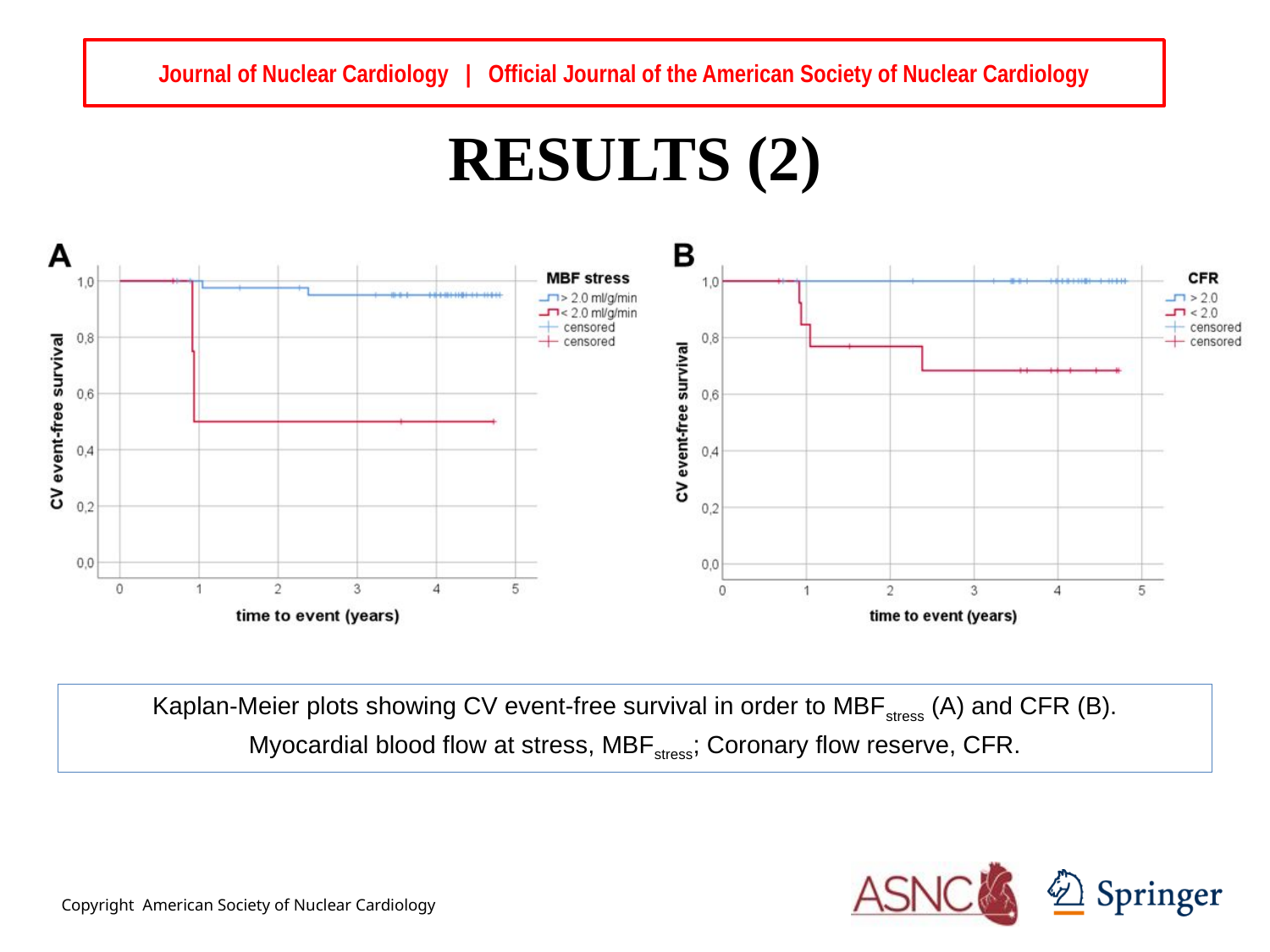

Journal of Nuclear Cardiology | Official Journal of the American Society of Nuclear Cardiology
# RESULTS (2)
Kaplan-Meier plots showing CV event-free survival in order to MBFstress (A) and CFR (B).
Myocardial blood flow at stress, MBFstress; Coronary flow reserve, CFR.
Copyright American Society of Nuclear Cardiology

## Slide 6
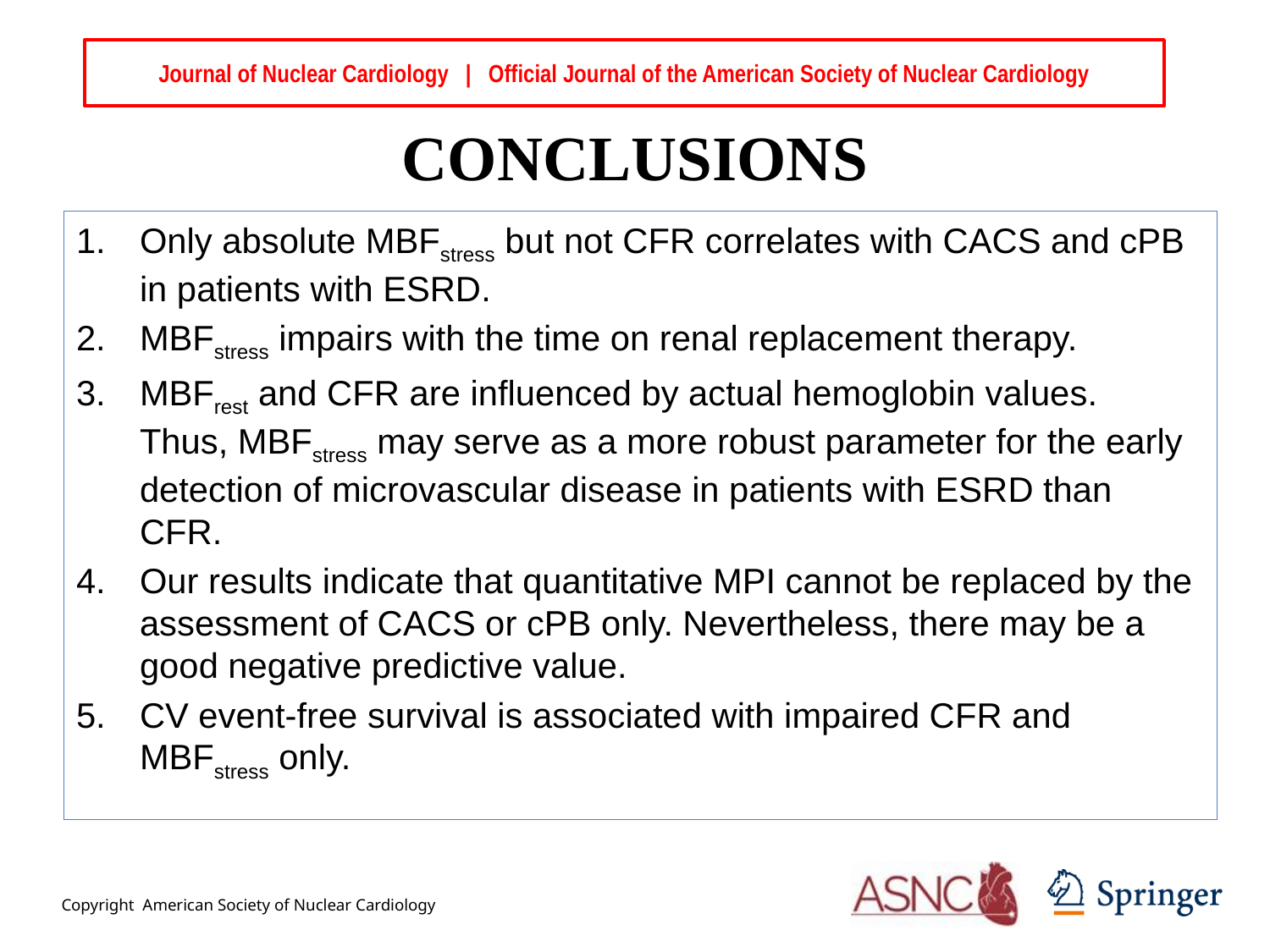

Journal of Nuclear Cardiology | Official Journal of the American Society of Nuclear Cardiology
# CONCLUSIONS
Only absolute MBFstress but not CFR correlates with CACS and cPB in patients with ESRD.
MBFstress impairs with the time on renal replacement therapy.
MBFrest and CFR are influenced by actual hemoglobin values.
Thus, MBFstress may serve as a more robust parameter for the early detection of microvascular disease in patients with ESRD than CFR.
Our results indicate that quantitative MPI cannot be replaced by the assessment of CACS or cPB only. Nevertheless, there may be a good negative predictive value.
CV event-free survival is associated with impaired CFR and MBFstress only.
Copyright American Society of Nuclear Cardiology
